# Expression, Characterization, Fermentation, Immobilization, and Application of a Novel Esterase Est804 From Metagenomic Library in Pesticide Degradation

**DOI:** 10.3389/fmicb.2022.922506

**Published:** 2022-07-07

**Authors:** Cuihua Chen, Gen Yu, Zhenyu Guo, Qihao Yang, Wenfeng Su, Qingfen Xie, Guandong Yang, Yifei Ren, He Li

**Affiliations:** ^1^Key Specialty of Clinical Pharmacy, The First Affiliated Hospital of Guangdong Pharmaceutical University, Guangzhou, China; ^2^Guangdong Key Laboratory of Pharmaceutical Bioactive Substances, College of Life Sciences and Biopharmaceuticals, Guangdong Pharmaceutical University, Guangzhou, China; ^3^CAS Testing Technical Services (Guangzhou) Co., Ltd., Guangzhou, China; ^4^Guangzhou Hua shuo Biotechnology Co., Ltd., Guangzhou, China

**Keywords:** pyrethroid pesticides, esterase, fermentation, immobilization, degradation

## Abstract

Esterase, as a type of powerful catabolic enzyme for the degradation of pyrethroid pesticides (PYRs), appears promising in improving the quality of crops and the environment contaminated by pesticide residues. The purpose of this research is to provide a detailed introduction to the enzymatic properties, optimal production and immobilization conditions, and the degradation ability of Est804 for PYRs. The study on enzymatic properties indicated that Est804 was an alkaline esterase with an optimal pH of 8.0 and a broad optimal temperature in the range of 35−50°C. The optimal activity of free Est804 was calculated to be 112.812 U, and the specific enzyme activity was 48.97 U/mg. The kinetic parameters of Est804 were *K*_m_ = 0.613 mM, *k*_cat_ = 12,371 s^–1^, and *V*_m_ = 0.095 mM/min. The results of the fermentative optimization demonstrated that the optimal conditions included 1.5% of inoculation amount, 30 mL of liquid volume, 28°C of the fermentation temperature, and 18 h of the fermentation time. The optimal medium consists of 15.87 g of yeast powder, 8.00 g of glycerol, and 9.57 g of tryptone in 1 L of liquid. The optimized enzyme activity was 1.68-fold higher than that before optimization. Immobilized Est804 exhibited the highest activity under the optimum preparation conditions, including 0.35 g of chitosan dosage, 0.4 mL of an enzyme, and 4 h at 40°C for adsorption. The degradation rates of Cypermethrin (CYP), fenpropathrin (FE), and lambda-cyhalothrin (LCT) by Est804 within 30 min were 77.35%, 84.73%, and 74.16%, respectively. The present study indicated that Est804 possesses great potential for the treatment of pesticide residues on crops and environmental remediation, conducive to the development of SGNH family esterase against pyrethroid accumulation.

## Introduction

Pyrethroid insecticides are a type of bionic synthetic insecticides based on natural pyrethrins and secreted from the plant *Chrysanthemum cinerariaefolium*, which possess the advantages of stable chemical properties, wide range of applications, strong potency in small doses, etc. Nowadays, they are widely used for pest control in agriculture, horticulture, forestry, and household worldwide (Chrustek et al., 2018; Bhatt et al., 2019). It has been reported that pyrethroids are approximately 2,250 times more toxic to insects than to mammals, and hence are gradually replacing organochlorine and organophosphate pesticides (OPs) (Chrustek et al., 2018; Bhatt et al., 2020). Pyrethroid insecticides were thought to exhibit very low toxicity and were even considered harmless to mammals (Chang et al., 2016); however, previous studies have demonstrated that pyrethroid metabolites may exert carcinogenic effects and cause contamination of various environmental compartments (Albaseer, 2019; Navarrete-Meneses and Pérez-Vera, 2019). Frequent applications of pyrethroids in agriculture and households will trigger inappropriate effects on human growth (Bhatt et al., 2019).

Pyrethroids kill insects by inducing perturbations of the voltage-sensitive sodium channel, which is a critical component of the insect nervous system (Scott, 2019). The pyrethroid insecticides can be divided into type I and type II based on their different chemical structures (Bhatt et al., 2020). Both type I and type II pyrethroids are able to kill a wide range of insects efficiently by contact, via producing prompt paralysis of the insect nervous system (Bhatt et al., 2019; Ramchandra et al., 2019). In addition to the lethal effect on insects, pyrethroid pesticides have been reported to negatively affect mammalian health by a number of studies. Type I pyrethroids, including permethrin, tetramethrin, and allethrin, act on the sodium channels in nerve filaments leading to a tremor type syndrome, which is characterized by several symptoms, such as tremors, aggressive behavior, hypersensitivity, and ataxia in mammals (Chrustek et al., 2018; Hołyńska-Iwan and Szewczyk-Golec, 2020). Different from type I, type II pyrethroids have an α-cyano group at the phenoxybenzyl moiety (Awoyemi et al., 2019). It has been reported that type II pyrethroids are more toxic to mammals than type I forms, and they cause salivation, incoordination, dermal tingling, and seizures when they act as gastrointestinal and central nervous system toxins (Bashir et al., 2013). Compared with type I pyrethroids, the presence of a cyano group in type II pyrethroids enhances their insecticidal properties (Bhatt et al., 2019), which might be the reason why type II forms were more toxic to mammals than type I forms.

To boost crop yield, different pesticides have been applied widely to control pests (Mao et al., 2020). At the same time, for the sake of the development of the economy and maintaining the health of mankind, highly toxic herbicides, such as paraquat, have been banned or replaced with pyrethroid insecticides. Hence, biological approaches have been proposed to eliminate the residues produced by pyrethroid insecticides that continually affect human health and their dwelling environment based on the characteristics of the pesticides. Among others, environmental bioremediation has emerged as a hotspot in the studies focusing on countering pyrethroid toxicity to marine life and mammals (Bhatt et al., 2019; Zhan et al., 2020). Pyrethroid-degrading strains and enzymes, as the means of bioremediation, have been proven to be an effective way of remediating pyrethroid-contaminated environments (Zhan et al., 2020).

Esterase has been identified as a powerful enzyme to degrade PYRs (Bhatt et al., 2020). Therefore, searching esterases for the efficient degradation of PYRs is of great significance. From the metagenomic library constructed by our team in the early stage, a gene of 804 bp in full length was obtained and named as *est804*. By sequencing and phylogenetic analysis, the hydrolase enzyme Est804 was identified as the esterase of the SGNH family. Besides the bioinformatic analysis of Est804, the prokaryotic expression, zymological properties, optimization of fermentation condition and immobilization, and application of esterase 804 were also investigated to determine the characteristics, improve the yield, and widen the applications of Est804. The results of our studies demonstrated that Est804 performed well in degrading PYRs and possessed good potential for the treatment of pesticide residues on crops and environmental remediation. Meanwhile, the degradation ability of PYRs by recombinant SGNH esterase Est804 was also studied in detail, which contributed to the development of SGNH family esterase against pyrethroid accumulation.

## Materials and Methods

### Biological Materials and Chemicals

The cloning host was *Escherichia coli* (*E. coli*) DH5α (Invitrogen, Carlsbad, CA, United States), the expression host was *E. coli* BL21 (DE3) (Novagen, Madison, WI, United States), and the tool used for protein expression was pET-28a (+) (Novagen). Markers, T4 DNA ligases, and all the restriction enzymes were purchased from TaKaRa (Dalian, China). DNA gel recovery kit and plasmid extraction kit were purchased from OMEGA (Norcross, GA, United States). Chemical reagents purchased from Sigma-Aldrich (St. Louis, MO, United States) were of analytical or electrophoresis grade.

### Bioinformatic Analysis

The gene *est804* was derived from the microbial metagenomic library of the soil samples collected from the south margin of Zhungeer Basin (45°40′N, 85°30′E). After genome sequencing, the sequence data of *est804* was deposited in the nucleotide sequence databases (GenBank) under accession number MW727216 and was also uploaded to NCBI^1^ to generate the corresponding amino acid sequence which was then compared to those of other esterase families using MEGA 7.0 software to construct the phylogenetic tree for investigating the relationship between Est804 and the esterase of other families. In addition, the homologous sequences of Est804 were found in different protein databases of NCBI. Using Clustal X 2.1 to complete the multiple sequence alignment, the result was uploaded to ESPript 3.0 combined with the three-dimensional structure of Est804 predicted on Phyre2 to generate an intuitive and clear map for the alignment of Est804 and other sequences, so the location of the conserved domain of the target esterase can be identified and analyzed.

### PCR Amplification and Construction of Prokaryotic Expression System

The gene *est804* was amplified from the pET-28a (+) plasmid by PCR, and the product was purified. The PCR primers used for the amplification were *est804*-F: (5′-CGCGGATCCATGGGATCCGAACATCATC-3′) and *est804*-R: (5′-CCCAAGCTTTTATGTGGACGCCTTGACAC-3′). The two underlined sequences display the recognition sites of restriction enzymes *BamH* I and *Hind* III, respectively. Hence, *est804*-pET-28 (+) was digested with *BamH* I and *Hind* III enzymes at 30°C and kept overnight. The resulting fragments of enzyme digestion and the pET-28a (+) vectors were ligated together using T4 DNA Ligase (Takara) in the water bath equipment at 16°C overnight. The recombinant plasmid was then introduced into *E. coli* BL21 (DE3) treated with calcium ions. The white monoclones were randomly selected after the incubation of the transformants became visible on the LB medium. The plasmids of the recombinant strain were extracted by restriction enzyme digestion as mentioned above, and electrophoresis was performed to verify whether the clones were positive. The successfully transformed clones were then inoculated in LB liquid medium and cultured until the OD_600nm_ of the bacterial suspension was between 0.6 and 0.8. The bacterial suspension was mixed with 20% glycerol in equal proportion and stored at −80°C in reserve.

### Verification of the Molecular Mass of Protein of Est804

With His-tag Protein Purification Kit (Beyotime, China), the purified protein of Est804 was prepared after collecting the culture broth under the optimum induction conditions. Staining with 10X loading buffer from Takara, the purified protein was denatured by exposing it to a high temperature (100°C) for 5 min. The molecular mass of protein Est804 was determined by sodium dodecyl sulfate-polyacrylamide gel electrophoresis (SDS−PAGE) at room temperature using 15% polyacrylamide gel and protein markers (TaKaRa) as standards.

### Enzymatic Assays

Esterase, as a kind of hydrolase enzyme, acts on the ester bonds of acetic acid and 4-nitrophenyl ester to produce p-nitrophenol (ρNP) that shows a maximum absorption peak at 405 nm. Thus, the activity of the esterase can be accessed by measuring the optical density at 405 nm (OD_405nm_). The inactive enzyme was treated with high temperature and acted as a negative control. A solution containing 0.1 M stock liquor was prepared by adding 36.2 mg of ρ-nitrophenyl acetate (ρNPA) into the EP tube containing 2 mL of methanol and mixing thoroughly to dissolve. The working solution was the mixed liquor consisting of 0.2 mL of 0.1 M stock solution and 4.8 mL of Britton-Robison (BR) buffer (pH 6.8). Then, 10 μL of diluted enzyme solution was added to 190 μL of the working solution and incubated in the water bath equipment at 40 °C for 20 min in triplicate. The standard curve of ρNP is shown in Supplementary Figure 1. Combined with the standard curve of BSA (Supplementary Figure 2), the specific activity of Est804 can be also calculated. All measurements were done in triplicate. One enzyme unit was defined as the amount of esterase required to liberate 1 μmol of ρNP from ρNPA per minute, and the total enzyme activity was expressed as 1 U. Relative enzyme activity was used as the index of experimental results under standard conditions. The highest enzyme activity was set as 100%, and the enzyme activity and stability were compared at different temperatures and pH values. After recording the OD_405nm_ of mixed reaction fluid, the total enzyme activity was calculated as follows:


(1) $$\mathrm{Enzyme}\,\mathrm{Activity}\,\left(U\right)=\frac{\left(181.2\,x-11.31\right)\times 0.2\times N}{0.01\times 20}$$

where x is the difference in the values of absorbance between experimental groups and control groups at 405 nm, N is the dilution multiple of enzyme solution (*N* = 1), 0.2 is the total volume of the reaction fluid (mL), 0.01 is the volume of enzyme liquid (mL) added to the reaction system, and 20 is the time of reaction (min).

### Determination of Kinetic Parameters

The kinetic parameters of free Est804 were determined at the standard conditions with 0.0−5.0 mM ρNPA as the substrate whose concentrations are varied at regular intervals. The experimental group with the final substrate concentration of 0.0 mM was used as the negative control, and the plot of Lineweaver–Burk double reciprocal was constructed to calculate the affinity constant (*K*_m_), the turnover number (*k*_cat_), and maximal velocity (*V*_max_) of Michaelis–Menten equation (Johnson and Goody, 2011).

### Optimization of Fermentation Conditions for the Recombinant Strain of Est804

To improve the yield of the novel esterase, the fermenting conditions for the recombinant strain were optimized. Four physicochemical factors were evaluated to determine which one significantly affected the shaking flask fermentation for the recombinant strain Est804. The independent variables included time, temperature, inoculation amount, and medium volume. The factors and levels for the L_9_ (3^4^) orthogonal experiment are presented in Table 1. In this study, we also investigated the combination of the components of auto-induced fermentation medium (AIFM), referring to the strategy and therapy established by Studier (2005). The Plackett–Burman (PB) experimental design (Table 2) was used to evaluate the relative importance of the different nutrient components by performing 12 experiments for Est804 production in submerged fermentation. The variables were designated and used with the high (+) and low (-) concentrations. The parameters tested included glycerin, yeast powder, tryptone, ammonium chloride, magnesium sulfate, phosphate, and microelement. The yield of Est804 was regarded as the dependent variable (Table 3), and three components of AIFM showing the greatest influence were selected according to the results of the PB experiment, evaluated by Box–Behnken design (BBD) (Table 4), and the optimized experimental results were statistically analyzed by using Design-Expert 8.0.6 software.

**TABLE 1:** Orthogonal experiment to determine the effect of independent factors on fermentation.

Level factor	1	2	3
A-Time (h)	14	16	18
B-Temperature (°C)	25	28	30
C-Inoculation Amount (%)	1.5	2	2.5
D-Medium Volume (mL)	20	30	40

**TABLE 2:** Parameters used for screening with Placket–Burman design.

Exp. No.	A	B	C	D	E	F	G	Relative enzyme activity (%)
1	1	1	−1	−1	−1	1	−1	92.35
2	1	−1	−1	−1	1	−1	1	76.43
3	−1	−1	1	−1	1	1	−1	72.79
4	−1	1	1	1	−1	−1	−1	81.77
5	1	1	−1	1	1	1	−1	97.28
6	1	−1	1	1	−1	1	1	85.49
7	−1	−1	−1	−1	−1	−1	−1	61.47
8	1	−1	1	1	1	−1	−1	82.17
9	−1	1	1	−1	1	1	1	88.76
10	−1	1	−1	1	1	−1	1	84.13
11	1	1	1	−1	−1	−1	1	100
12	−1	−1	−1	1	−1	1	1	67.78

**TABLE 3:** Plackett–Burman design for screening of parameters for Est804 production in fermentation.

Label	Parameter	Unit	Low level (−1)	High level (−1)
A	Glycerin	g/L	6	10
B	Yeast Powder	g/L	10	20
C	Tryptone	g/L	5	15
D	Ammonium Chloride	g/L	2	4
E	Magnesium Sulfate	mM	4	8
F	Phosphate	mM	25	75
G	Microelement	mM	1	2

**TABLE 4:** Three levels for components of auto-induced fermentation medium (AIFM).

Label	Factor	Unit	−1	0	1
A	Yeast Powder	g/L	10	15	20
B	Glycerin	g/L	4	8	12
C	Tryptone	g/L	5	10	15

### Immobilization of Est804

Since the enzymes are highly specific to the substrate type, even under mild conditions of temperature, pressure, and pH, the bioenzymes exhibit a better and more efficient catalytic effect than conventional chemical catalysts (Filho et al., 2019). To enhance the stability and the ability of biocatalysis recycling of Est804, chitosan microspheres were used as the immobilization materials. The preparation process for immobilized enzyme was similar to a previous report (Wang et al., 2019), but with certain modifications as follows: 1 g of chitosan was added to 50 mL of 1% acetic acid solution, and the mixture was stirred evenly before keeping it at 37°C overnight to ensure that the bubbles were completely dissolved and disappeared. With the dripping of the mixture into the fixator, chitosan microspheres of the same size were gradually formed. After soaking for 3 h, the chitosan carrier was filtered out from gauze, and the residual fixative was rinsed with ddH_2_O. After drying completely at room temperature, chitosan microspheres of a certain mass were weighed and added into 0.5 mL of crude enzyme solution for adsorption at low temperature for 6 h. After the removal of the unabsorbed free enzyme, total enzyme activity and immobilized enzyme activity were measured according to the aforementioned standard method of enzymatic assays. The calculation formula for the recovery rate of enzyme activity is:


(2) Immobilizedenzymeactivityrecovery(%)=

$$\,\frac{\mathrm{Immobilized}\,\mathrm{enzyme}\,\mathrm{activity}}{\mathrm{Total}\,\mathrm{enzyme}\,\mathrm{activity}}\times 100\%$$

(Kumaret al., 2019)

### Enzymatic Degradation of Pyrethroid Pesticides

The degradation ability of PYRs by recombinant Est804 was determined. Cypermethrin (CYP), fenpropathrin (FE), and lambda-cyhalothrin (LCT), which are frequently used for agricultural products and environments (Cámara et al., 2020; Rebouillat et al., 2021; Xiao et al., 2021), were used as the degradation substrates, and GC-2010PLUS gas chromatography equipment produced by Shimadzu Company in Japan was used for the quantitative analysis. The standard mixture of CYP, FE, and LCT was prepared with a BR buffer solution of pH 7.5 to obtain a final concentration of 6 mg/L. About 4 mL of crude enzyme solution was added to 1 mL of the prepared standard mixture of pesticides, and the group of inactivated enzyme solutions was regarded as a negative control. About 1 μL of standard sample solution was used to determine the peak area of three pesticides, and the standard curves of the PYRs-peak area were constructed (Supplementary Figure 3). The values of R^2^ show that the mass concentration of three types of PYRs is linearly correlated with the peak area in the range of 0.025 to 0.4 mg/L. The reaction of the mixed solution was conducted at 37°C for 30 min. About 10 mL of hexane and 5 g of NaCl were added into 5 mL of the reaction solution to be tested at constant temperature and shaking at 180 rpm for 30 min. After centrifugation at 10,000 rpm for 4 min, 1 μL of the reaction solution was taken for quantitative analysis by gas chromatography. The injection conditions were as follows: HP-5 (30 m × 0.25 mm × 0.25 μm), non-split injection, 1.0 μL of injection volume, 280°C of injector temperature, 2.0 mL/min of column flow, high purity nitrogen as carrier gas at 16.7 mL/min of total flow rate, 300°C of ECD detector temperature, 0.05 nA of electric current, and 30 mL/min of make-up gas flow rate. The oven temperature was maintained at 150°C for 1 min, then increased to 220°C at a rate of 20°C/min for 10 min, and to 270°C at a rate of 20°C/min for 9.5 min. All the tests were performed five times.

## Results and Discussion

### Analysis for Biological Information of Est804

Since culture-dependent techniques provide limited insights into the microbial diversity of the pyrethroid-polluted environments, and therefore genomics and metagenomics approaches are widely adopted in search of novel esterase. In this study, the gene *est804* of carboxylesterase from the metagenomic library constructed by our team in the early stage was discovered and studied. The results of Open Reading Finder (ORF) on NCBI presented that gene *est804* was the gene with a total length of 804 bp encoding 267 amino acids. The analysis of the conserved domain (CD) search indicated that Est804 belongs to the SGNH hydrolase superfamily (Supplementary Figure 4). The active sites of Est804 resemble the typical Ser-His-Asp (Glu) triad from other serine hydrolases. According to the results of BLASTP (Supplementary Figure 5), the order of protein with the top three scores presented that the amino acid sequence of Est804 is 75.10% identical to the hypothetical protein FOQG_18516 obtained from *Fusarium oxysporum* (Gen Bank: EXK76751.1) and shares 74.69% identity with the hypothetical protein Forpi1262_v011166 from *Fusarium oxysporum* (Gen Bank: KAG7427934.1). The highest percent identity between Est804 and F53441_6683 (Gen Bank: KAF4450165.1) was found to be 77.59%.

### The Construction and Analysis of Phylogenetic Tree of SGNH Est804

Various esterases can be divided into different families according to their biological characteristics. There are 18 esterase families that have been identified, and many more are being discovered (Shuang, 2020). In order to classify Est804, a phylogenetic tree was constructed starting with 28 sequences representing the identified bacterial hydrolase family, which shows how Est804 is placed against the genes from 18 esterase families (Supplementary Figure 6). Except for Est804, other amino acid sequences were retrieved from GenBank. The analysis of the phylogenetic tree shows that the amino acid sequence of Est804 shows a certain evolutionary distance from all known esterases of other families. Combined with the results of the BLASTP query (Supplementary Figure 5), it could be inferred that SGNH Est804 is a novel esterase.

### Multiple Sequence Alignment for Analyzing Genetic Characterization of Est804

**FIGURE 1:**
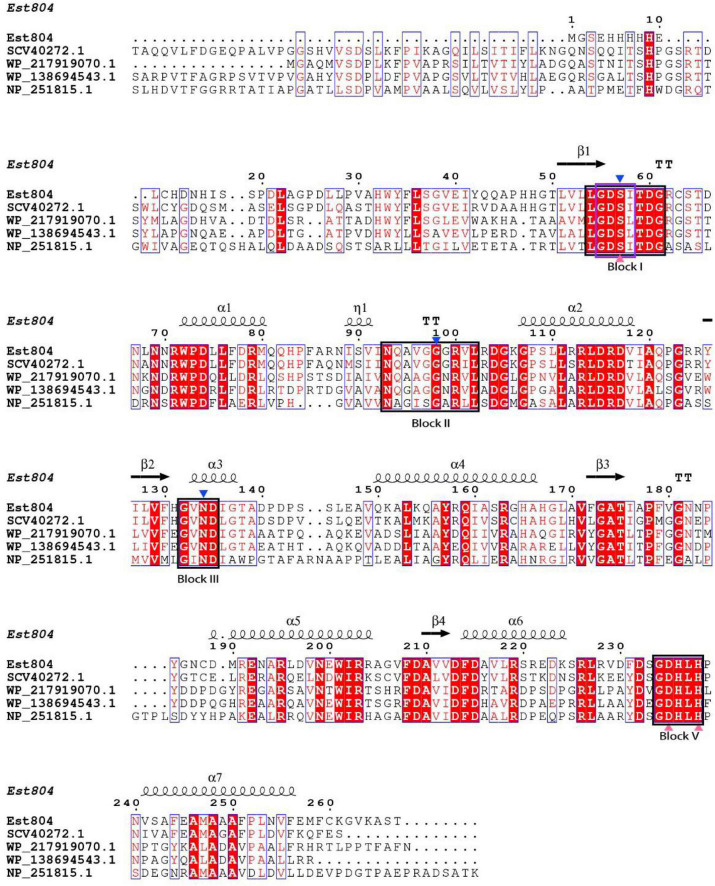
Multiple sequence alignment of Est804 with the esterase of SGNH/GDSL family. Apart from the amino acid sequence of Est804 (this work), the sequences of SGNH hydrolase from *Fusarium fujikuroi* sp. B14 (SCV40272.1), SGNH/GDSL hydrolase from *Actinomadura* sp. BRA 177 (WP_217919070.1), SGNH/GDSL hydrolase from *Nonomuraea zeae* (WP_138694543.1), and hypothetical protein of SGNH family from *Pseudomonas aeruginosa* PAO1 (NP_251815.1) are derived from GenBank database in NCBI. Four highly or absolutely conserved domains are presented in the black boxes (Block I, II, III, and V). The typical conserved domain G-D-S-X of SGNH hydrolase family is shaded by a purple box in Block I. The blue-filled down triangles indicate oxyanion hole residues. The catalytic triad of Est804 consisting of Ser, His, Asp is labeled by pink-filled triangles. The secondary element assignment, above the alignment, corresponds to the structure of Est804. Black helices represent α-helices and black arrows represent the β-strands.

As Figure 1 shows, the structure of Est804 predicted by Phyre2 showed that Est804 belongs to an α/β hydrolase, owing to its clear folding structure composed of seven α-helix and four β-sheets. The multiple sequence alignment with other esterases revealed that Est804 had a typical conserved motif G-D-S-X (marked in the purple box) and a catalytic triad identified as Ser57-Asp235-His238 (labeled with pink-filled triangles). According to the annotated information of four known proteins in GenBank and combining with the comparison results by Clustal X 2.1, we found that the oxyanion hole residues of Est804 were Ser57, Gly98, and Asn134. The presence of Asp at the third amino acid preceding His in Block V indicated that DxxH serves as the third member of the catalytic triad, and this is the same as TAP (Akoh et al., 2004).

### Expression and Purification of SGNH Family Esterase Est804

As Supplementary Figure 7A shows, the specific bands of PCR amplification products appeared at the location between 750 and 1,000 bp, indicating that the length of the products is consistent with the predicted size of the target gene fragment. The band of 804 bp (Lane 1, Supplementary Figure 7A) in electrophoresis was cut off and purified after double enzyme digestion using *Bam* I and *Hind* III enzymes. The linked products of Est804 and pET-28a(+) (Lane 2, Supplementary Figure 7B) were introduced into E. coli BL21 (DE3), and the recombinant bacteria were inoculated on the LB medium with 100 μg/mL of kanamycin at 37°C till the clones appeared. The plasmid of clones was then extracted, and the expression products were digested by *Bam* I and *Hind* III to verify whether the transformation was positive. The electrophoresis result of the double enzyme digestion products of positive transformants is shown in Lane 1 (Supplementary Figure 7B). The recombinant Est804 was easily produced in the LB medium at pH 7.0 with the induction of 0.08 mmol/L IPTG at 30°C and incubation for 10 h. Uninduced and optimally induced bacterial fluids were separately collected to prepare the samples for protein electrophoresis according to the described method. In Supplementary Figure 8, it is apparent that the location of the target protein of recombinant Est804 was between 26 kDa and 34 kDa, that is, about 30 kDa, which is in agreement with the theoretical prediction of 29.61 kDa by using ExPASy ProtParam tool.

### Kinetic Parameters of SGNH Est804

**FIGURE 2:**
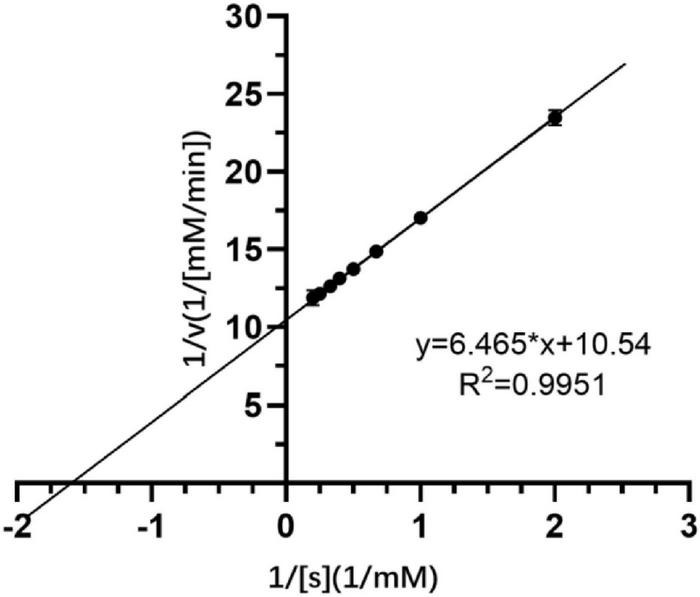
Kinetic parameters of SGNH Est804.

Crude extracellular SGNH Est804 protein obtained from the supernatant after the cell lysis of the recombinant strain showed great activity toward ρNPA among the substrates with other fatty acid chains. Therefore, using ρNPA as a substrate, the kinetic parameters of Est804 were studied and measured under the standard conditions. The kinetic parameters of Est804 were *K*_m_ = 0.613 mM, *k*_cat_ = 12,371 s^–1^, and *k*_cat_/*K*_m_ = 20,181 mM^–1^s^–1^. According to the formula shown in Figure 2, the V_*max*_ of Est804 was calculated to be 0.095 mM/min. Compared with PE8 obtained from the marine bacterium *Pelagibacterium halotolerans* B2^T^ whose *K*_m_ was 0.83 mM (Wei et al., 2013) and EstA from *Streptomyces lividans* TK24 whose *k*_cat_/*K*_m_ was 2296.14 mM^–1^s^–1^(Chang et al., 2021), Est804 exhibited a greater affinity for ρNPA. The subsequent studies on the properties of Est804 were carried out using ρNPA as the substrate.

### Effect of Temperature and pH on Activity and Stability of Est804

**FIGURE 3:**
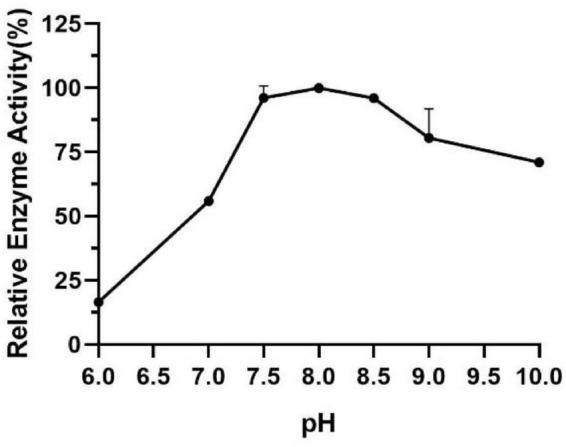
Effect of pH on the activity of SGNH Est804.

**FIGURE 4:**
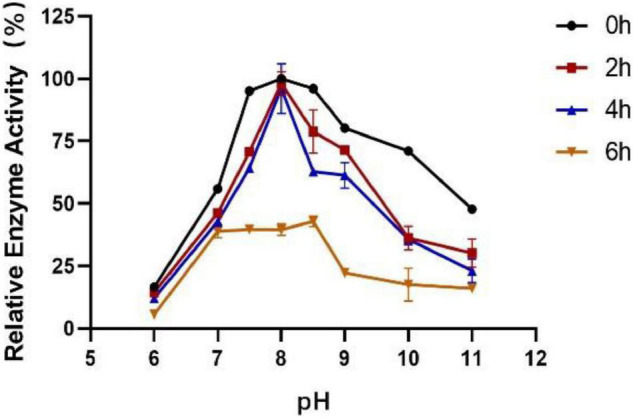
Effect of pH on the stability of SGNH Est804.

The influence of pH on the enzyme activity of Est804 was tested between pH 6.0 and pH 10.0 at 40°C. As Figure 3 shows, the enzyme activity of Est804 increased significantly from pH 6.0 to pH 8.0, particularly in the range of pH 6.0–7.5. Similar to Tlip from *Thauera* sp. (Yu et al., 2018), Est804 reached the highest enzyme activity when the pH of the solution was 8.0. The total enzyme activity of Est804 at pH 8.0 was calculated to be 104.054 U, and the specific activity was 45.17 U/mg, which is nearly 9-fold higher than that of EstHE1 (5.6 U/mg) (Okamura et al., 2010). Obviously, Est804 retained more than 70% of the highest activity in the solution of pH 8.0 to 10.0, indicating that Est804 has a good tolerance for the alkaline environment. The stability of Est804 for 0∼6 h in the solution of different pH values is shown in Figure 4. The result shows that the relative enzyme activity of SGNH Est804 decreased by only 3.9% after being held in the solution of pH 8.0 for 4 h, which illustrates that the storage stability of SGNH Est804 in the solution of pH 8.0 is much better than the stability in other pH solutions. However, the relative enzyme activity of Est804 decreased significantly between pH 6.0 and pH 10.0 after 6 h, suggesting that the enzyme retained relatively high activity when stored in the solution of pH 8.0 for no more than 4 h.

**FIGURE 5:**
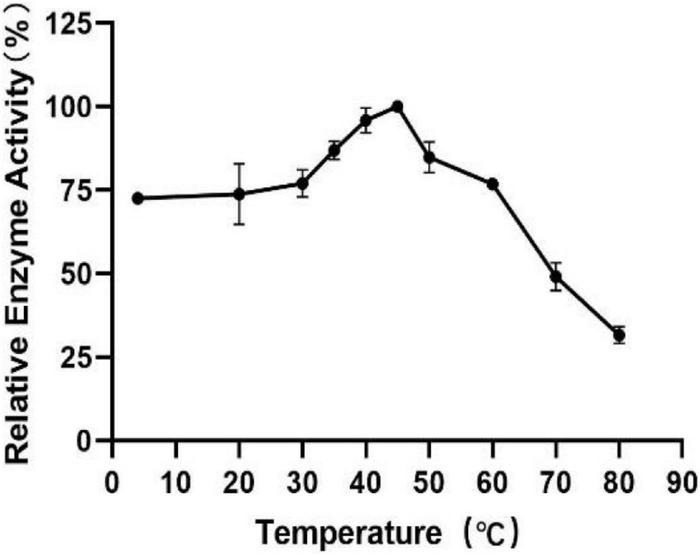
Effect of temperature on the activity of SGNH Est804.

**FIGURE 6:**
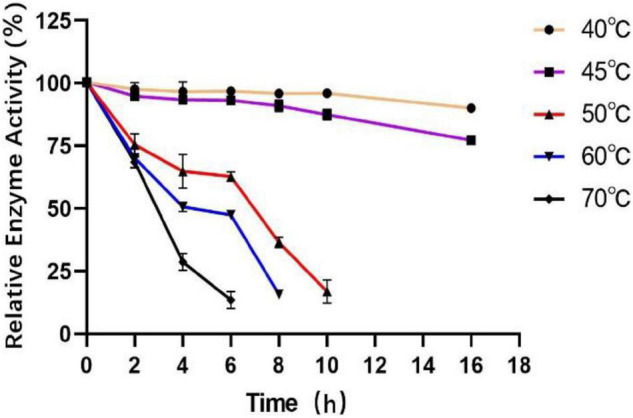
Effect of temperature on the stability of SGNH Est804.

To measure the optimal temperature of Est804, the catalytic activity was assessed in the solution of pH 8.0 at different temperatures from 4°C to 80°C, and the whole trend is shown in Figure 5. The enzyme activity obviously decreased when the temperature was above 45°C. When the temperature was in the range of 4°C∼30°C, the enzyme activity of Est804 increased gradually in the range of 72−75%, while increased rapidly over 30°C and reached the highest enzyme activity at 45°C, and this finding is similar to PE8 with ρNPA as the substrate (Wei et al., 2013). The total enzyme activity of Est804 at 45°C was 112.812 U, nearly 8 U higher than that observed in the solution at pH 8.0 without temperature optimization. With a specific activity of 48.97 U/mg at the optimal temperature, Est804 exhibited a close enzyme activity to EstA whose specific enzyme activity was 47.9 U/mg toward ρNPA2 (Chang et al., 2021). The broad optimal temperature in the range of 35−50°C indicated that Est804 is capable of exhibiting excellent hydrolytic activity at a moderate temperature range. The thermal stability of Est804 for 0−16 h at 40°C, 45°C, 50°C, 60°C, and 70°C is shown in Figure 6. The trend of yellow and purple lines indicates that Est804 possessed good thermostability at 40°C and 45°C for 10 h. The enzyme activity at 40°C and 45°C was found to be 95.95% and 87.48%, respectively. After being held at 50°C for 6 h, 60°C for 4 h, and 70°C for 2 h, Est804 still exhibited above 50% of the maximum activity. The analysis of the effect of temperature on thermostability showed that Est804 had a good thermal tolerance at a moderate temperature range and its optimal temperature for storage is 40°C.

### Optimization for the Fermentation of Recombinant Strain Est804

**FIGURE 7:**
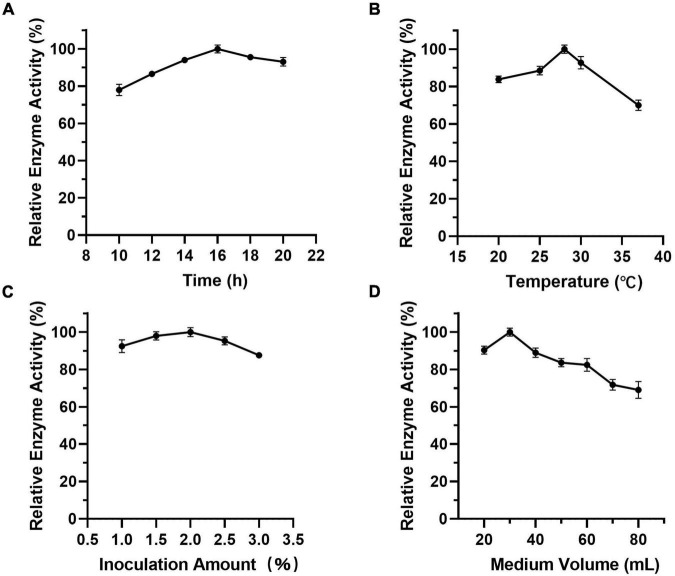
**(A-D)** Effect of factors on the activity of Est804.

As Figure 7 shows, when the culture time was 16 h (Figure 7A), the temperature was 28°C (Figure 7B), the inoculation amount of recombinant strain was 2.0% (Figure 7C), and the medium volume was 30 mL (Figure 7D), the activity of Est804 reached the highest. Based on the investigation of the effect of single factors on enzyme activity, the L_9_ (3^4^) orthogonal test was used to determine the factors for optimal fermentation conditions of Est804 recombinant strain, so as to establish the optimum fermentation process of enzyme production. The results presented in Table 5 showed that the esterase (Est804) generated by the recombinant strain exhibited the highest enzyme activity when the fermentation conditions were similar to No.2 experiment, namely A_1_B_2_C_2_D_2_, that is, 14 h of fermentation time, 28°C of culture temperature, 2% of inoculation amount, and 30 mL of medium volume. The residual analysis presented that the order of the influence of each factor on the test index was liquid medium volume, temperature, time, and inoculation amount. The statistical significance of Eq. (3) was tested by the F test, and the results of the analysis of variance (ANOVA) are summarized in Table 6:


(3) R=82.54+6.42⁢A+8.18⁢B+2.63⁢C+0.57⁢D+1.06⁢E

+1.54⁢F+1.23⁢G

**TABLE 5:** Orthogonal experimental design, L_9_ (3^4^): effect of time (A), temperature (B), inoculation amount (C), and liquid medium volume (D) on the shaking flask fermentation of recombinant strain Est804.

Exp. No.	A	B	C	D	Relative enzyme activity (%)
1	1	1	1	1	93.34
2	1	2	2	2	100
3	1	3	3	3	85.35
4	2	1	2	3	88.35
5	2	2	3	1	96.32
6	2	3	1	2	94.23
7	3	1	3	2	96.85
8	3	2	1	3	94.11
9	3	3	2	1	92.53
K1	92.897	92.847	93.893	94.063	
K2	92.967	96.810	93.627	97.027	
K3	94.497	90.703	92.840	89.270	
R	1.600	6.107	1.053	7.757	
Order of Factor Preference	RD > RB > RA > RC				
Optimal Level	A3	B2	C1	D2	

**TABLE 6:** Variance analysis for optimal components of liquid medium (R^2^ = 0.9870).

Parameter	Sum of squares	Degree of freedom	Mean square	*F*-value	*P*-value	Ranking of significance
Model	1444.12	7	206.30	43.24	0.0013	*
A	494.34	1	494.34	103.62	0.0005	2
B	802.95	1	802.95	168.30	0.0002	1
C	82.90	1	82.90	17.38	0.0140	3
D	3.88	1	3.88	0.81	0.4184	7
E	13.44	1	13.44	2.82	0.1686	6
F	28.46	1	28.46	5.97	0.0710	4
G	18.15	1	18.15	3.81	0.1229	5
Residual	19.08	4	4.77			
Cor. Total	1463.20	11				

The *P*-value equal to 0.0013 indicates that the probability that the result was due to error is 0.13%. It can be seen from Table 6 that the seven media components that affect the fermentation of bacterial strains are yeast powder, glycerin, tryptone, phosphate, trace elements, magnesium sulfate, and ammonium chloride in the descending order of influence. Three of the most important components of AIFM affecting enzyme production were selected as the factors for the BBD experiment (Table 7), which were as follows: A-glycerin (P = 0.0005), B-yeast powder (P = 0.0002), and C-tryptone (P = 0.0140). Thus, the optimal prediction model of enzyme production (Table 8) was obtained:


(4) Enzyme⁢activity=-286.38917+112.25426⁢A+272.64909⁢B

+311.54378⁢C+1.85079⁢A⁢B-1.48707⁢A⁢C

$$+9.77698\,B\,C-3.55351\,A^{2}-24.72410\,B^{2}$$

$$-19.11943\,C^{2}$$

**TABLE 7:** Box–Behnken test for enzyme production of AIFM.

Exp. No.	A	B	C	Relative enzyme activity%	Enzyme production (U/L)
1	0	−1	1	64.12	2063.87
2	0	0	0	99.23	3193.98
3	0	0	0	100	3218.76
4	0	−1	−1	79.02	2543.46
5	1	0	−1	86.88	2796.46
6	0	1	1	76.53	2463.32
7	0	1	−1	67.13	2160.75
8	1	1	0	85.96	2766.85
9	0	0	0	98.87	3182.39
10	0	0	0	97.96	3153.10
11	1	0	1	78.04	2511.92
12	−1	0	1	77.89	2507.09
13	0	0	0	98.14	3158.89
14	1	−1	0	82.63	2659.66
15	−1	−1	0	83.92	2701.18
16	−1	1	0	82.65	2660.31
17	−1	0	−1	82.11	2642.92

**TABLE 8:** Analysis of variance for Est804 production (R^2^ = 0.9942, Adj R^2^ = 0.9868).

Factors	Sum of variance	Degrees of freedom	Mean square	*F*-value	*P*-value
Model	2.01E+06	9	2.23E+05	133.74	<0.0001
A-Yeast Powder	6237.44	1	6237.44	3.74	0.0942
B-Glycerin	862.04	1	862.04	0.52	0.4952
C-Tryptone	44611.12	1	44611.12	26.78	0.0013
AB	5480.66	1	5480.66	3.29	0.1126
AC	5528.42	1	5528.42	3.32	0.1113
BC	1.53E+05	1	1.53E+05	91.81	<0.0001
A^2^	33230.11	1	33230.11	19.95	0.0029
B^2^	6.59E+05	1	6.59E+05	395.55	<0.0001
C^2^	9.62E+05	1	9.62E+05	577.49	<0.0001
Residual	11660.54	7	1665.79		
Lack of Fit	8797.96	3	2932.65	4.1	0.1033
Pure Error	2862.58	4	715.65		
Cor Total	2.02E+06	16			

**FIGURE 8:**
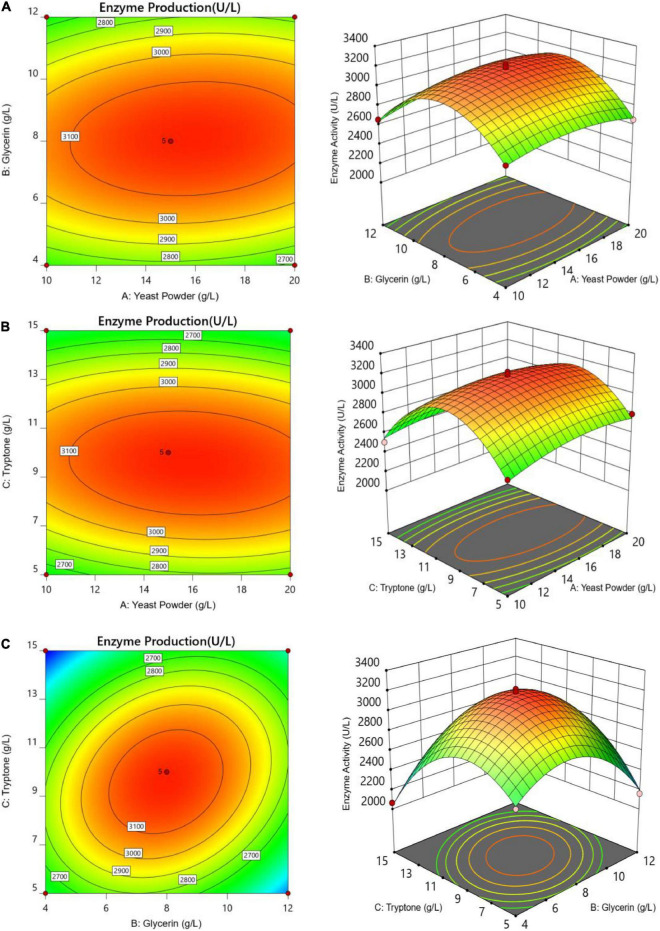
Response surface and contour maps of the influence of panels **(A)** yeast powder, **(B)** glycerin, and **(C)** tryptone on AIFM for enzyme production.

The letters A, B, and C in the formula refer to yeast powder, glycerin, and tryptone, respectively. The model fitted well with practical results, and the accuracy was 99.42% for the analysis and prediction of fermentation medium components for the recombinant strain. Figure 8 demonstrates the response surface curve and contour diagram of the effects of three components on bacterial fermentation in AIFM. The optimal concentrations of the three factors were 15.87 g/L of yeast powder, 8.00 g/L of glycerin, and 9.57 g/L of tryptone. Under the optimal experimental conditions, the yield of an esterase by recombinant strain reached 3,215.78 U/L, which was close to the predicted value of 3,187.03 U/L, indicating that the predicted model was reliable. The enzyme activity after optimal fermentation was 1.68-fold higher than that without optimization.

### Enzymatic Properties of Immobilized Enzyme

**FIGURE 9:**
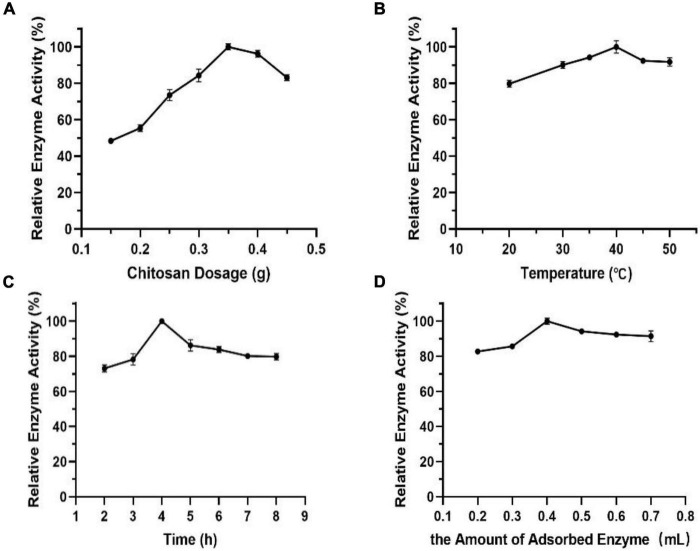
Effect of panels **(A)** chitosan dosage, **(B)** temperature, **(C)** time, and **(D)** the amount of adsorbed enzyme on immobilization.

It can be seen from Figure 9A that the relationship between chitosan dosage and enzyme activity shows approximately a normal distribution. The enzyme activity of immobilized Est804 reached the maximum value when the chitosan dosage was 0.35 g and declined above 0.35 g. It was speculated that the decrease was due to the competitive adsorption of enzyme protein among chitosan carriers, which resulted in the destruction of protein structure and the decline of enzyme activity. In addition to chitosan dosage, other factors, including temperature, time, and the amount of adsorbed enzyme, closely related to immobilized enzyme activity were also investigated. In terms of the effect of temperature on immobilization, a part of the adsorbed enzyme is easily lost during the filtration process because of the unstable combination of enzyme and carrier at lower temperature, and the adsorption capacity of the carrier to enzyme changes significantly under the influence of structure variance caused by high temperatures. The effect of time on immobilization is no less important for immobilization than that generated by temperature. The combination between enzyme and carrier will not be sufficient if the immobilization time is too short, while the enzyme activity does not increase in extremely long time periods. The fourth factor to be considered is the amount of adsorbed enzyme. Partial chitosan would fail to complement the satisfying adsorption due to lack of enzyme amount or excess enzyme was added. The results indicated that the optimal temperature, time, and the most appropriate amount of adsorbed enzyme for immobilization is 40°C (Figure 9B), 4 h (Figure 9C), and 0.4 mL, respectively (Figure 9D).

### Comparison of Biochemical Characterization Between Free and Immobilized Est804

**FIGURE 10:**
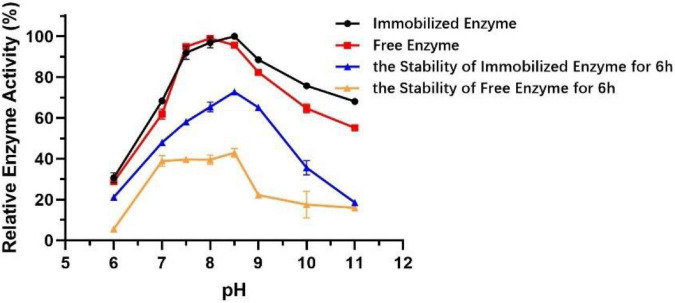
Effect of pH on the activities and stability of immobilized esterase Est804.

The technology of enzyme immobilization, which involves the physical adsorption of a particular enzyme to proper support by covalent bonds, provides a favorable solution for the production of immobilized enzymes, which show better activity, broader pH range, and thermal stability compared to free enzymes (Rafiee and Rezaee, 2021). Different from that of free enzyme, the optimum pH of immobilized Est804 increased from 8 to 8.5, and the stability of immobilized enzyme was greatly improved in the solution at pH 7.5−10 (Figure 10). After being held in the solution of pH 7.5−9 for 6 h, the activity of immobilized Est804 still remained above 50% of the maximum enzyme activity.

**FIGURE 11:**
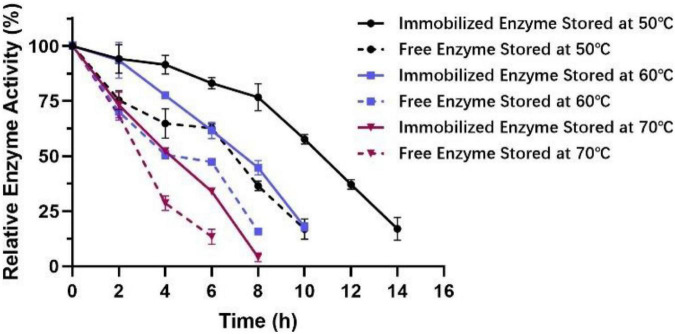
Effect of temperature on the stability of immobilized esterase Est804.

It can be seen from Figure 11 that the activity of immobilized enzymes relative to that of the free enzymes increased from 50°C to 70°C. At the same time, the stability of the immobilized enzyme was significantly enhanced in the high-temperature environment of 50−70°C for 4−6 h. Remarkably, at 50°C, the enzyme activity still remained above 50% of the maximum activity after 10 h, indicating that the tolerance of Est804 for moderate temperature had been greatly improved after immobilization.

### Storage and Recycle Stability of Immobilized Est804

About 6.0 g of immobilized Est804 was stored at 4°C and 25°C, respectively, and 0.35 g of immobilized enzyme was taken every 1.5 days to determine the enzyme activity. It is obvious that no matter it is storged in free or immobilized form, the enzyme activity of Est804 always decreases with the increasing of its exsisting time according to the results of Figure 11. However, as Tale 9 shows, the activity of immobilized Est804 could still maintain 91.35% of the highest enzyme activity at 4°C and 85.27% of the highest enzyme activity at 25°C over 9 days, indicating that immobilized Est804 possessed good storage stability.

**TABLE 9:** Storage stability of immobilized Est804.

Storage time (d)	Relative enzyme activity (%)	
	
	4°C	25°C
0	100	100
1.5	98.12	99.46
3.0	97.91	97.94
4.5	96.82	95.85
6.0	95.43	95.16
7.5	93.29	91.18
9.0	91.35	85.27

It has been reported that immobilization permits the reuse of enzymes and continuous operation, which results in the improvement of the process economy (Patel et al., 2016; Matsumoto et al., 2019). The common methods for immobilization are physical adsorption, covalent binding, encapsulation, and crosslinking. The immobilization yields lower relative activity, as the loading of the enzyme on the supports tends to change significantly with different immobilization methods. Physical adsorption involves a simple operation and is less expensive, but it can easily desorb enzymes due to the dependence on the weak force between enzymes and the carrier (Akbar et al., 2015). Covalent binding usually requires a modifiable and highly stable carrier with more chemical groups, and the operation process may be complex (Akbar et al., 2015). The advantage of encapsulation is that the enzyme molecules are fixed on the grid structure of the porous carrier and catalyze the reaction under mild reaction conditions, which is in line with the requirements of the enzyme (Akbar et al., 2015). However, the substrate can hardly react with the enzyme because of the influence of the embedding structure. Crosslinking effectively improves enzyme leakage, but the immobilized enzyme prepared by simple crosslinking possesses poor mechanical properties, so it is usually used in combination with other methods to strengthen the effect of immobilization (Akbar et al., 2015). Taken together, there are pros and cons of each method used for immobilization. In this work, the activity of the immobilized enzyme in the seventh recycle decreased by 63.12% compared to that in the first cycle, but it should be taken into account that it was the result of adsorption only through physical interaction avoiding some significant impacts brought by other methods. The decrease in the activity of the immobilized enzymes can result from every operation of elution procedure before reuse. Table 10 illustrates that immobilized Est804 still remained more than 70% of the maximum enzyme activity after it was repeatedly used less than 5 times, proving that chitosan microspheres maintained good activity of Est804 and greatly contributed to the repeated capability of Est804 for hydrolysis against ester substrate.

**TABLE 10:** Recycle stability of immobilized Est804.

Times of recycle	Relative enzyme activity (%)
1	100
2	97.19
3	86.35
4	78.87
5	72.45
6	53.69
7	36.88

### Degradation of Three Pyrethroids by Est804

**FIGURE 12:**
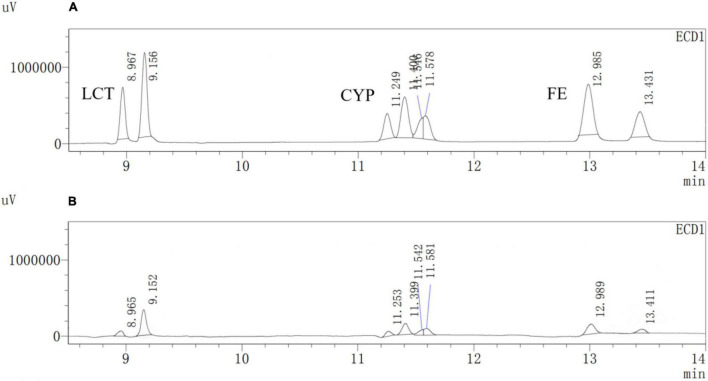
GC analysis of the degradation of Cypermethrin (CYP), fenpropathrin (FE), and lambda-cyhalothrin (LCT) by Est804. **(A)** CG; **(B)** EG.

A previous study reported that PYRs are able to penetrate the crop matrix and possibly get converted to toxic oxidation and hydrolysis products for human intake (Wu et al., 2007). Esterase provides a promising way to solve such a potential problem brought by the residual PYRs on crops, which can be attributed to the fact that pyrethroids are the ester-containing compounds whose main degradation route involves cleavage of the ester bond (Sogorb and Vilanova, 2002). In the process of improving pesticide degradation, some researchers tried to isolate strains that produced hydrolases with degradation ability from the environment where pyrethroids were accumulated. However, most wild-type bacteria have the disadvantages of low degradation efficiency for pyrethroid pesticides and a long fermentation period. For example, it is reported that the degradation rate of CYP in the mineral salt medium by *Brevibacillus parabrevis* JCm4 and *Pseudomonas aeruginosa* JCm8 selected from agricultural soil by enrichment culture within 10 days was nearly 28.0% and 46%, respectively (Akbar et al., 2015). Merely 12.6% degradation of FE was achieved in a mineral salt medium by *Clostridium* sp. ZP3 obtained from effluent in a pesticide factory following 12 days (Zhang et al., 2011). About 56% of LCT was degraded by *Catellibacterium* sp. CC-5 isolated from contaminated soil within 7 days (Zhao et al., 2013). Hence, looking for or excavating microorganisms that generate esterases within a short fermentation period and exhibit high degradation efficiency for pyrethroid pesticides has become an urgent need. Est804 from the SGNH family showed a wide range of substrate properties and certain degradation ability for PYRs, which could be verified by the results of GC analysis. The recovery experiments were carried out after the standard mixture of three PYRs with six concentrations ranging from 0.06 to 1.50 mg/L was prepared. As shown in Supplementary Table 1, the average recoveries of CYP, FE, and LCT were 93.5%∼106.5%, 91.5%∼109.3%, and 92.8%∼109.5%, respectively. The relative standard deviations (RSDs) of all tests were within 4%, proving that the precision of the detection meets the requirement of analysis. The experiment was divided into a control group (CG) and an experimental group (EG), as shown in Figure 12. The degradation rate of CYP, FE, and LCT are calculated according to the peak area and the standard curve formulas (Supplementary Figure 3). The present work aims at simulating the degradation process of the PYR residues on crops having a tiny number of residual pesticides still existing (Albaseer, 2019), and thus 0.2 mg/L of PYRs was chosen to be the substrate concentration for the reaction mixture to be treated with enzyme solution. As Table 11 shows, the degradation rates of CYP, FE, and LCT by Est804 within 30 min were 77.35%, 84.73%, and 74.16%, respectively. However, the results of our research cannot be directly compared with those of the recent studies on the capacity of PYR degradation by other isolated strains, since the experiments were carried out under the abiotic conditions over a short period of time, but it provides favorable support for developing enzyme preparations that can be industrially produced. According to the study reported by Birolli et al. (2021), only 16.7% CYP degradation was determined under abiotic conditions, whereas 36.6 ± 1.9% biodegradation was observed for *B. thuringiensis* Berliner with the native microbiome. The example evidently indicated that bioaugmentation with the bacterial strain promoted a significant increase in the pesticide decontamination, prompting that Est804 can be used in biological conditions to enhance the degradation of pyrethroids, which is possibly of great benefit for application in agricultural residue treatment and environmental remediation.

**TABLE 11:** Degradation rate of Cypermethrin (CYP), fenpropathrin (FE), and lambda-cyhalothrin (LCT) by SGNH family Est804.

PYRs	Initial concentration (mg/L)	The concentration after degradation (mg/L)						Degradation rate (%)
			
		1	2	3	4	5	Mean	
CYP	0.207572	0.045	0.042	0.047	0.049	0.052	0.047	77.35%
FE	0.209650	0.031	0.034	0.032	0.028	0.036	0.032	84.73%
LCT	0.209011	0.062	0.052	0.048	0.051	0.057	0.054	74.16%

## Conclusion

Considered to be a safer alternative to OPs, the application of PYRs has been significantly increased (Cycon and Piotrowska-Seget, 2016; Albaseer, 2019). The widespread and continuous use of PYRs creates the problem of polluting the terrestrial and aquatic environments and affecting non-target organisms (Cycon and Piotrowska-Seget, 2016), implying the importance of their removal from crops and environment. In order to promote the degradation of PYRs, discovering efficient esterases for the treatment of pesticide residues and environmental remediation is of utmost importance. In this study, the gene *est804* from the metagenomic library constructed by our team in the early stage was discovered and studied. Encoded by the gene with a total length of 804 bp, SGNH Est804 is a novel carboxylesterase that exhibits the highest activity in the alkaline environment (pH = 8.0) at a moderate temperature (45°C). After immobilization, the tolerance of Est804 to an alkaline environment and a higher temperature was improved, displaying better stability than the free form after storge over the same time period. In addition, the immobilized Est804 also provided good reusability for hydrolysis against ester substrates. In addition to investigating the enzymatic characteristics of Est804, our research also confirmed that Est804 possessed great degradation ability for three types of PYRs, including CYP, FE, and LCT, within a short time after the verification by GC analysis. Only a few studies have reported on the degradation of PYRs by esterase encoded by the genes selected from the metagenomic library. Our investigation of the novel carboxylesterase Est804, which shows a broad spectrum and efficient degradation ability, not only fills the gap of the lack of studies on functional genes encoding hydrolases for the biodegradation of PYRs, but also provides favorable support for the development of the SGNH family esterase against pyrethroid accumulation.

## Data Availability Statement

The original contributions presented in this study are included in the article/Supplementary Material, further inquiries can be directed to the corresponding author.

## Author Contributions

CC Contributed the conceptualization and original draft preparation. GYu and ZG edited the manuscript. QY, WS, and QX reviewed the manuscript. GYa and YR supervised the data. HL carried out the project administration and supervision. All authors contributed to the article and approved the submitted version.

## Conflict of Interest

The authors declare that the research was conducted in the absence of any commercial or financial relationships that could be construed as a potential conflict of interest.

## Publisher’s Note

All claims expressed in this article are solely those of the authors and do not necessarily represent those of their affiliated organizations, or those of the publisher, the editors and the reviewers. Any product that may be evaluated in this article, or claim that may be made by its manufacturer, is not guaranteed or endorsed by the publisher.

## Footnotes

1
https://www.ncbi.nlm.nih.gov/
