# Supplementary material for: Expression, Characterization, Fermentation, Immobilization, and Application of a Novel Esterase Est804 From Metagenomic Library in Pesticide Degradation
Source: Front Microbiol. 2022 Jul 7;13:922506. doi: 10.3389/fmicb.2022.922506 (PMC9301488; doi:10.3389/fmicb.2022.922506)
Supplement: Supplementary file 1 [file Data_Sheet_1.docx]

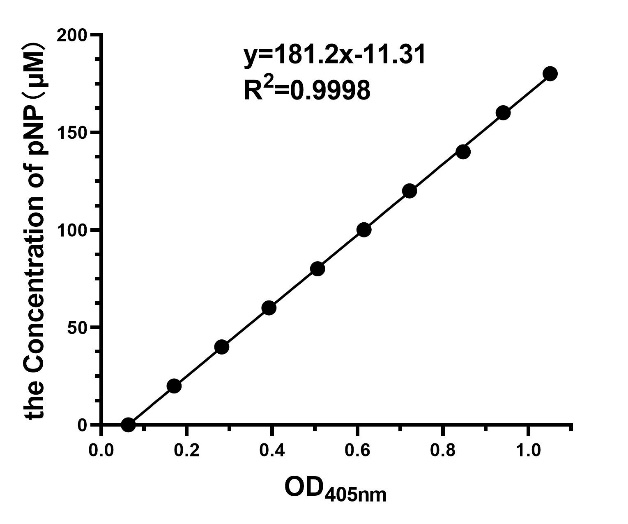


Supplementary Figure 1 the Standard Curve of pNP


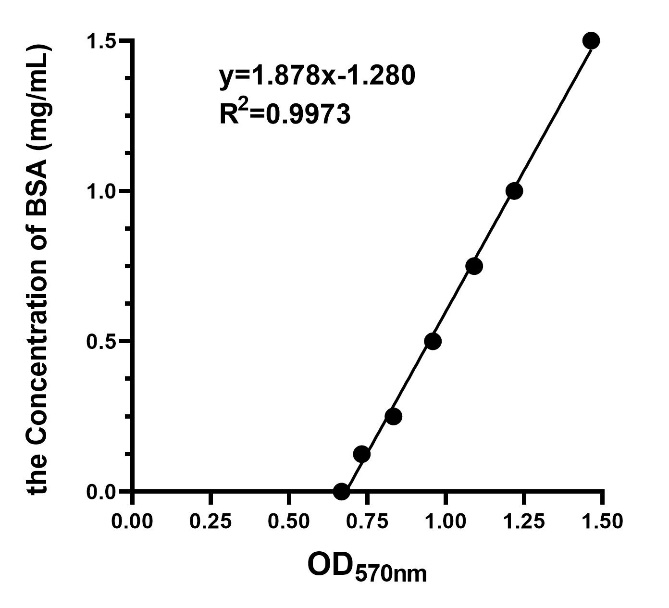


Supplementary Figure 2 the Standard Curve of BSA

**(A)**
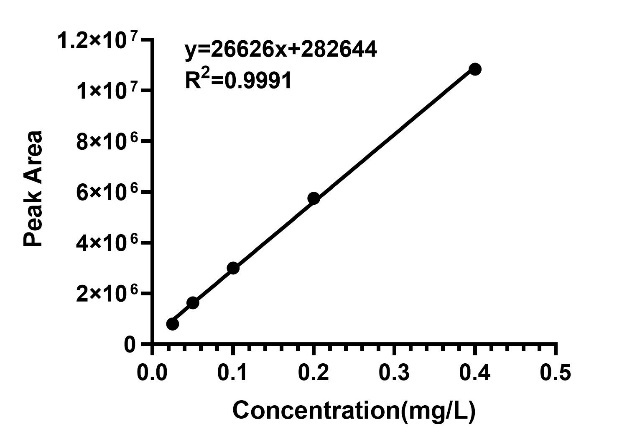


**(B)**
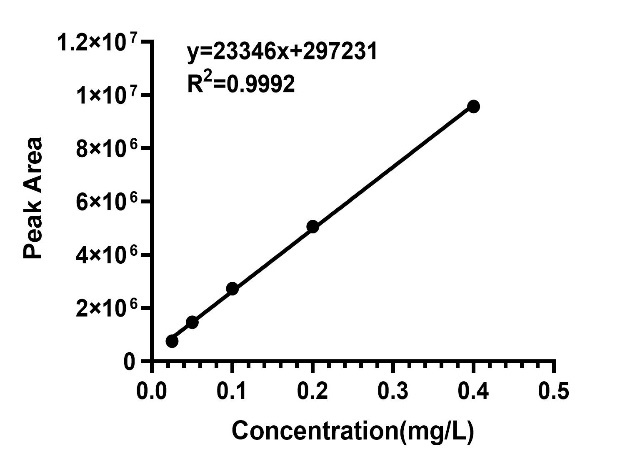


**(C)**
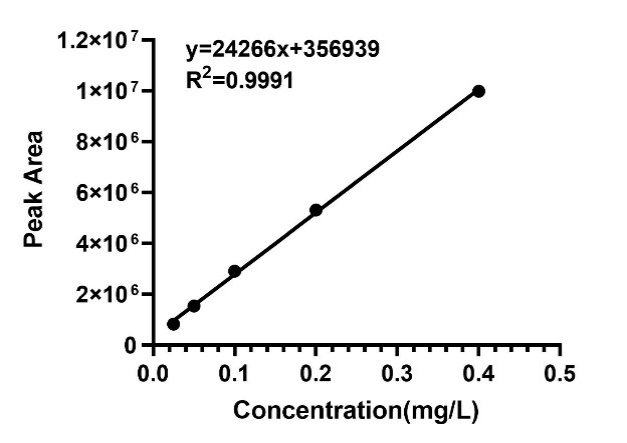


Supplementary Figure 3 the Standard Curve of (A)CYP, (B)EF, and (C)LCT


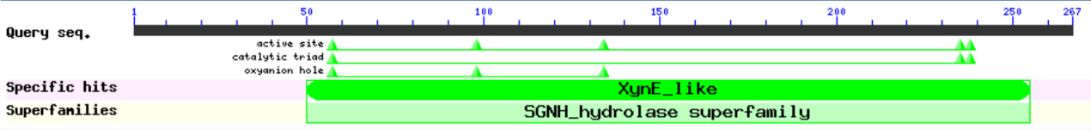


Supplementary Figure 4 Presumed Similar Domain Architectures of SGNH Esterase Est804


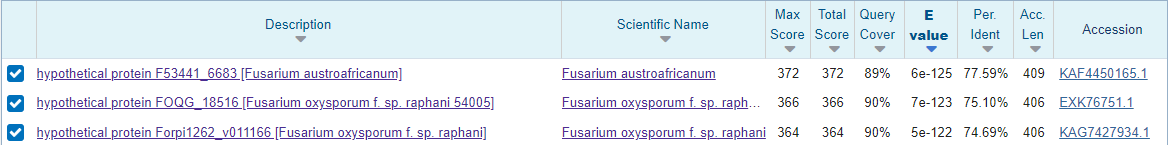


Supplementary Figure 5 the Presentation of Amino Acid Sequences with the Highest Scores by BLASTP for Est804


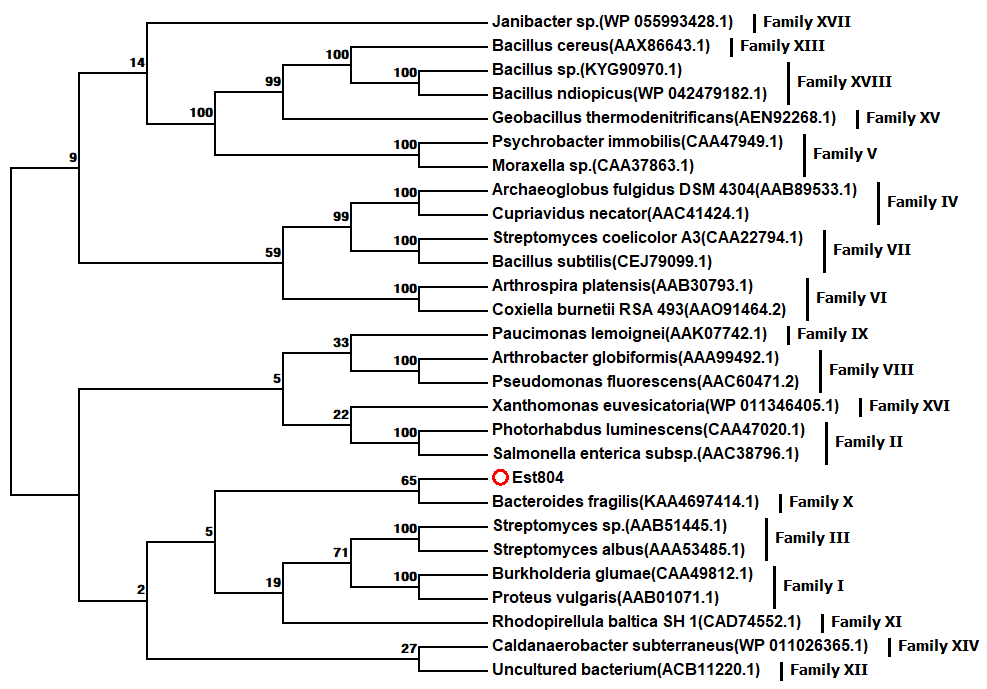


Supplementary Figure 6 Unrooted Bootstrap consensus Tree of Est804 and Closely Related Sequence from Eighteen Esterase Families Using Neighbor-Joining Method

Est804 was indicated by red circle. The esterase from other microorganisms were annotated with family classification by subheadings.


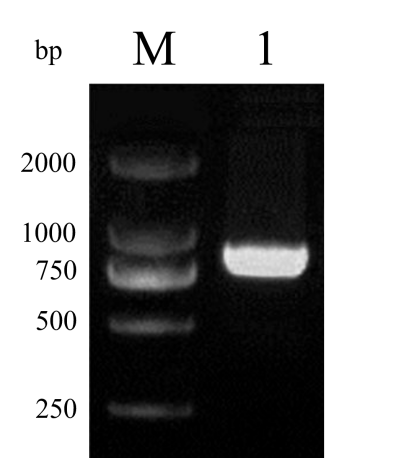


Supplementary Figure 7A Amplified Products of Gene Est804 Electrophoresis

M: DL2000 DNA Marker；Lane 1: Gene Est804


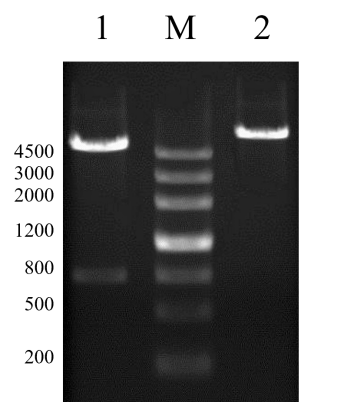


Supplementary Figure 7B Electrophoresis Identification of Recombinant Est804 and Digested Plasmids

M: DL4500 DNA Marker；Lane 1: the Enzyme-digested Products of pET-28a(+)-est804；Lane 2：pET-28a(+)-est804


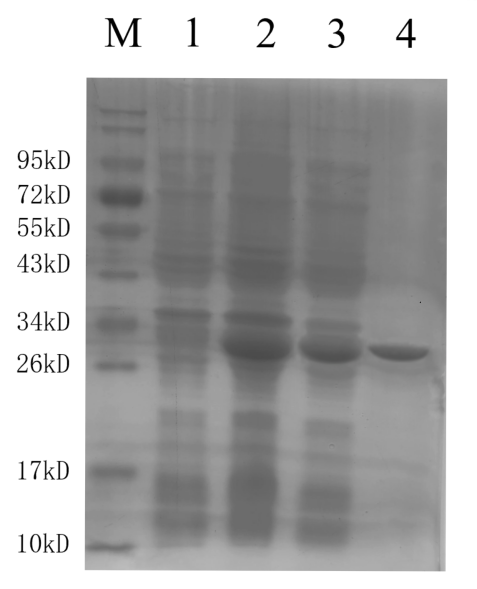


Supplementary Figure 8 SDS-PAGE Analysis of Recombinant SGNH Est804

M: Protein MW Marker；1: Uninduced Cell Lysis of Recombinant Est804；2: Induced Cell Lysis of Recombinant Est804；3: Induced Cell Lysis of Supernatant of Recombinant Est804；4: Purified Recombinant Est804
