# Supplementary material for: Expression, Characterization, Fermentation, Immobilization, and Application of a Novel Esterase Est804 From Metagenomic Library in Pesticide Degradation
Source: Front Microbiol. 2022 Jul 7;13:922506. doi: 10.3389/fmicb.2022.922506 (PMC9301488; doi:10.3389/fmicb.2022.922506)
Supplement: Supplementary file 2 [file Data_Sheet_2.docx]

**Supplementary material**

Protein sequence of Est804 (GenBank accession No. MW727216):

MGSEHHHHHELCHDNHISSPDLAGPDLLPVAHWYFLSGVEIYQQAPHHGT

LVLLGDSITDGRCSTDNLNNRWPDLLFDRMQQHPFARNISVINQAVGGGR

VLRDGKGPSLLRRLDRDVIAQPGRRYILVFHGVNDIGTADPDPSSLEAVQ

KALKQAYRQIASRGHAHGLAVFGATIAPFVGNNPYGNCDMRENARLDVNE

WIRRAGVFDAVVDFDAVLRSREDKSRLRVDFDSGDHLHPNVSAFEAMAAA

FPLNVFEMFCKGVKAST

Nucleotide sequence of Est804(GenBank accession No. MW727216):

ATGGGATCCGAACATCATCATCATCATGAACTATGCCACGACAATCACATCTCGTCTCCAGACCTTGCCGGTCCGGACTTGCTGCCGGTAGCGCATTGGTACTTTCTCTCTGGTGTCGAGATCTATCAACAAGCCCCCCACCATGGCACGCTTGTTCTTCTCGGTGATAGCATAACCGACGGCCGCTGCAGCACAGATAATCTCAACAACCGTTGGCCAGATCTACTCTTTGACCGAATGCAACAGCATCCTTTTGCGCGCAATATTTCTGTAATCAACCAGGCCGTGGGTGGCGGGAGAGTCCTCCGCGATGGCAAAGGACCAAGCCTTCTTCGTCGCCTGGATCGCGATGTCATCGCCCAGCCCGGCCGGCGCTACATCCTCGTCTTCCATGGTGTCAACGACATCGGCACAGCTGATCCTGATCCCTCGTCTCTAGAAGCAGTGCAGAAAGCTCTCAAGCAGGCCTATCGCCAGATTGCTTCACGAGGGCATGCACATGGTCTAGCTGTCTTTGGTGCAACAATTGCACCGTTCGTGGGAAATAATCCGTACGGGAATTGCGATATGCGCGAGAATGCGCGACTGGACGTCAATGAGTGGATTCGAAGAGCAGGTGTCTTTGACGCTGTGGTGGACTTTGATGCTGTACTACGGTCTAGGGAGGATAAGAGTCGTCTCCGGGTGGACTTTGACTCCGGAGATCATTTGCATCCAAACGTGTCAGCTTTTGAGGCTATGGCAGCAGCATTTCCTCTCAATGTGTTTGAGATGTTCTGCAAAGGTGTCAAGGCGTCCACATAA

**Supplementary information**

Supplementary Table 1 Basic Parameters of Gas Chromatography

| PYRs | Concentration of the Standard Mixture (mg/L) | Recovery(%) | RSD(%) |
| --- | --- | --- | --- |
| CYP | 0.06 | 106.5±3.7 | 2.4 |
|  | 0.12 | 101.7±5.1 | 3.5 |
|  | 0.24 | 97.8±3.9 | 2.8 |
|  | 0.48 | 101.2±4.8 | 2.5 |
|  | 0.96 | 96.8±4.4 | 2.2 |
|  | 1.50 | 93.5±6.1 | 3.3 |
| FE | 0.06 | 97.6±4.5 | 2.9 |
|  | 0.12 | 103.5±1.2 | 2.8 |
|  | 0.24 | 97.8±4.9 | 1.8 |
|  | 0.48 | 109.3±1.5 | 3.4 |
|  | 0.96 | 94.6±7.3 | 2.8 |
|  | 1.50 | 91.5±7.2 | 3.1 |
| _­_ LCT | 0.06 | 103.1±3.4 | 1.2 |
|  | 0.12 | 109.5±5.3 | 2.7 |
|  | 0.24 | 96.3±3.5 | 3.4 |
|  | 0.48 | 100.2±2.2 | 3.6 |
|  | 0.96 | 95.2±6.1 | 3.4 |
|  | 1.50 | 92.8±7.5 | 2.9 |
